# Redox properties and PAS domain structure of the *Escherichia coli* energy sensor Aer indicate a multistate sensing mechanism

**DOI:** 10.1016/j.jbc.2022.102598

**Published:** 2022-10-15

**Authors:** Zachary A. Maschmann, Teck Khiang Chua, Siddarth Chandrasekaran, Héctor Ibáñez, Brian R. Crane

**Affiliations:** Department of Chemistry and Chemical Biology, Cornell University, Ithaca, New York, USA

**Keywords:** redox sensing, flavoprotein, electron transport chain, energy taxis, transmembrane receptor, CW, clockwise, DDM, *N*-dodecyl-β-d-maltoside, ESR, electron spin resonance, ETC, electron transport chain, FAD, flavin adenine dinucleotide, FAD_ASQ_, FAD anionic semiquinone, FAD_HQ_, FAD hydroquinone, FAD_OX_, oxidized FAD, FL, full-length, FRE, NADH-dependent FAD oxidoreductase, HAMP, histidine kinases, adenylate cyclases, methyl-accepting chemotaxis proteins, and phosphatases, KCD, kinase control domain, LOV, light–oxygen–voltage, MCP, methyl-accepting chemotaxis protein, *Mm*ETF, *Methylophilus methylotrophus* electron-transferring flavoprotein, MSP1D1, membrane-scaffold protein D1, NSQ, neutral semiquinone, PAS, Per–Arnt–Sim, SAXS, small-angle X-ray scattering, SEC, size-exclusion chromatography, SHE, standard hydrogen electrode

## Abstract

The Per–Arnt–Sim (PAS; named for the representative proteins: Period, Aryl hydrocarbon receptor nuclear translocator protein and Single-minded) domain of the dimeric *Escherichia coli* aerotaxis receptor Aer monitors cellular respiration through a redox-sensitive flavin adenine dinucleotide (FAD) cofactor. Conformational shifts in the PAS domain instigated by the oxidized FAD (FAD_OX_)/FAD anionic semiquinone (FAD_ASQ_) redox couple traverse the HAMP (histidine kinases, adenylate cyclases, methyl-accepting chemotaxis proteins, and phosphatases) and kinase control domains of the Aer dimer to regulate CheA kinase activity. The PAS domain of Aer is unstable and has not been previously purified. Here, residue substitutions that rescue FAD binding in an FAD binding–deficient full-length Aer variant were used in combination to stabilize the Aer PAS domain. We solved the 2.4 Å resolution crystal structure of this variant, Aer-PAS-GVV, and revealed a PAS fold that contains distinct features associated with FAD-based redox sensing, such as a close contact between the Arg115 side chain and N5 of the isoalloxazine ring and interactions of the flavin with the side chains of His53 and Asn85 that are poised to convey conformational signals from the cofactor to the protein surface. In addition, we determined the FAD_ox_/FAD_ASQ_ formal potentials of Aer-PAS-GVV and full-length Aer reconstituted into nanodiscs. The Aer redox couple is remarkably low at –289.6 ± 0.4 mV. In conclusion, we propose a model for Aer energy sensing based on the low potential of Aer-PAS–FAD_ox_/FAD_ASQ_ couple and the inability of Aer-PAS to bind to the fully reduced FAD hydroquinone.

Motile bacteria correlate their swimming behavior with environmental signals by directly sensing small molecules with transmembrane chemoreceptors (1, 2, 3). Canonical dimeric chemoreceptors bind attractants and repellants in the periplasmic space and transduce conformational signals across the inner membrane to a dedicated histidine kinase CheA through the assistance of an adaptor protein CheW (4). Two trimers-of-receptor dimers associate with dimeric CheA and two subunits of CheW to form core signaling units, the minimal assembly state for transducing chemotactic signals (2, 5, 6). Core signaling units further assemble into extended arrays of hexagonal symmetry that enable signal gain and sensitivity to small changes in ligand concentration (4, 7). During chemotaxis, inhibition of CheA by attractants (kinase-off) causes counterclockwise flagellar rotation and smooth swimming, whereas activation of CheA by removal of attractants or addition of repellants causes clockwise (CW) flagellar rotation and cell tumbling. Closely related to chemotaxis is energy taxis, which is taxis in response to respiratory changes; in particular, to concentration changes of terminal electron acceptors of the electron transport chain (ETC), such as nitrate, fumarate, and especially oxygen (8, 9, 10). The sensing of oxygen, either directly through binding O_2_ or indirectly through energy sensing, is termed aerotaxis (10). Energy receptors monitor the cellular energy state and couple to the chemotactic signaling infrastructure to regulate motility (8, 9). Chemotaxis functions to situate respiring bacteria in microenvironments abundant in essential nutrients such as sugars and amino acids. As bacteria oxidize these nutrients, they deplete their surroundings of electron acceptors, and energy taxis is required to stimulate migration before energy depletion (or in the case of oxygen, anoxia) develops (11, 12). Chemotaxis and energy taxis cooperate to position bacteria in a microenvironment appropriate to their metabolic needs (11). Energy taxis allows cells to monitor the metabolic flux through the ETC, which in turn couples to the ADP/ATP ratio, the membrane potential (Δψ), the osmotic potential, ΔpH, and the redox states of ETC respiratory complexes (10). Mechanisms of energy sensing correspondingly vary with respiratory requirements (8, 9, 10).

The primary energy sensor for motility in *Escherichia coli* is the dimeric transmembrane chemoreceptor Aer (aerotaxis receptor), which responds to changes in oxygen concentration indirectly by monitoring the metabolic load of reducing equivalents in the ETC (13, 14). Aer-mediated taxis has been shown to correlate with NADH dehydrogenase activity, and Aer sensitivity to respiratory flux is increasingly thought to be achieved through interactions with respiratory complexes (15).

Aer is dimeric and composed of a cytoplasmic N-terminal PAS (Per–Arnt–Sim; named for the representative proteins: Period, Aryl hydrocarbon receptor nuclear translocator protein and Single-minded) domain, a helical transmembrane domain, a cytoplasmic HAMP (histidine kinases, adenylate cyclases, methyl-accepting chemotaxis proteins [MCPs], and phosphatases) domain, and a C-terminal coiled-coil kinase control domain (KCD) with high homology to those of MCPs (16) (Fig. 1*A*). Aer responds to oxygen indirectly through monitoring the metabolic flux of the ETC (10, 13, 14). In reconstitution studies, Aer associates into higher order signaling units like other *E. coli* MCPs such as Tar and Tsr, though no direct evidence of Aer receptor clustering has been reported in cells (17). Homologs of *E. coli* Aer are also found in pathogenic bacteria such as *Pseudomonas putida* and *Campylobacter jejuni* (18, 19, 20). Motility-coupled respiration is a major virulence determinant of *Salmonella enterica typhimurium*, a prominent foodborne pathogen (21).

**Figure 1:**
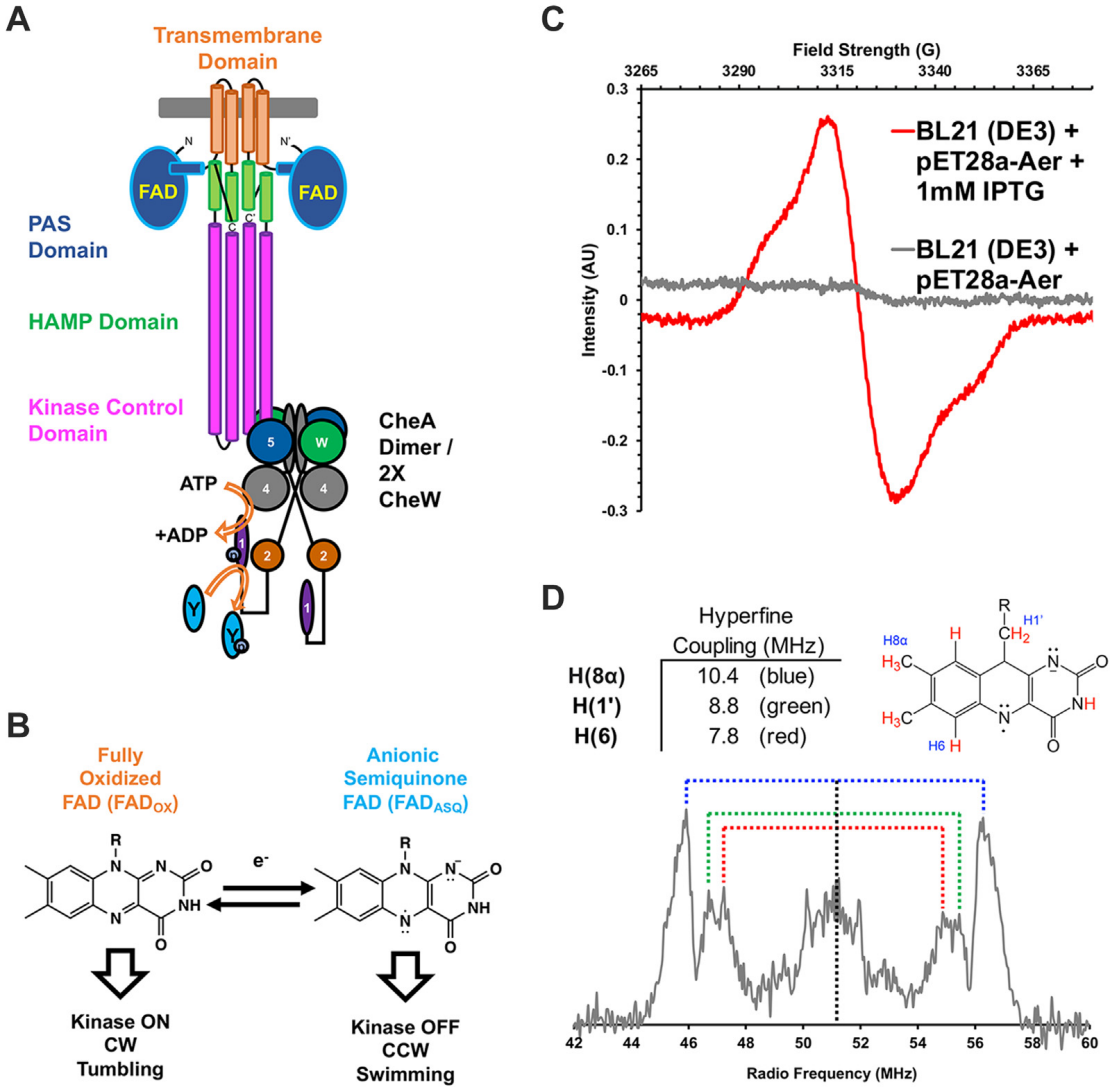
***Escherichia coli* Aer domain arrangement, cofactor chemistry, and function.***A*, cartoon of an Aer dimer (the cytoplasmic PAS domain: *blue*, the transmembrane region: *orange*, the HAMP domain: *green*, and the kinase control domain: *magenta*). Each PAS domain binds an FAD cofactor. The protein interaction region (PIR) of the kinase control domain contacts and regulates autophosphorylation of the dimeric histidine kinase CheA in concert with the coupling protein CheW (W). CheA, whose domains are labeled 1 to 5, transfers phosphate to a secondary messenger CheY that affects flagellar rotation directly. *B*, scheme showing the redox chemistry of FAD bound to Aer. Fully oxidized FAD bound to Aer undergoes a one-electron reduction to anionic semiquinone FAD (FAD_ASQ_). The redox state of FAD determines Aer signaling output (below). *C*, the FAD cw-ESR signal from Aer WT expressed in BL21/DE3 cells from the pET-28a expression plasmid before and after induction of growth using 1 mM IPTG. *D*, *in vivo* ENDOR spectrum of Aer WT in BL21/DE3 cells, consistent with FAD_ASQ_. A schematic of the FAD isoalloxazine ring with the relevant hydrogen atoms referenced to the relevant hyperfine couplings (indicated by color in the *in vivo* ENDOR spectrum). FAD, flavin adenine dinucleotide; HAMP, histidine kinases, adenylate cyclases, methyl-accepting chemotaxis proteins, and phosphatases; PAS, Per–Arnt–Sim.

The sensory module of Aer is the intracellular PAS domain that binds a redox-sensitive flavin adenine dinucleotide (FAD) prosthetic group. The redox state of PAS-FAD determines the Aer signaling output. The flavin undergoes changes in redox state that trigger conformational transitions within the PAS domain that propagate to the KCD to transduce Aer signaling events (13, 17, 22). Reconstituted into nanodiscs, fully oxidized FAD (FAD_OX_) confers a kinase-on signaling state, and the FAD anionic semiquinone (FAD_ASQ_) confers a kinase-off signaling state (Fig. 1*B*) (17). Certain mutations alter cofactor-coupled Aer signaling; for example, the Aer Y111C variant produces an inverted response in signal output relative to FAD redox state (13). Aer-dependent aerotaxis relies on the ability of Aer to switch signaling states; mutations that confer a constitutive kinase-on or kinase-off signal allow motility but do not support energy taxis (14). Determining the reduction potential of Aer-bound FAD is critical for understanding Aer redox sensitivity and for identifying ETC species capable of interacting with Aer. *E. coli* cells switch from a kinase-on (CW) to a kinase-off (counterclockwise) state when (1) electron donors are introduced to reduce an oxidized ETC or (2) electron acceptors are introduced to oxidize a reduced ETC. Aer activity in nanodiscs is then consistent with this behavior because, as noted previously, Aer FAD_OX_ produces kinase-on and Aer FAD_ASQ_ produces kinase-off. It follows that the fully reduced FAD hydroquinone (FAD_HQ_), which would be favored under highly reducing conditions, should also produce kinase-on (CW). However, the FAD_HQ_ state has not yet been observed for the isolated protein, even upon reduction with dithionite or incubation with reduced FAD_HQ_; hence, the reduction potential of the Aer FAD_ASQ_ must be very low (17, 23). PAS–HAMP interactions are necessary to stabilize the PAS domain in the full-length (FL) protein, and the PAS domain alone has therefore never before been purified successfully (17, 23, 24). Conformational transitions internal to the PAS domain following FAD reduction promote the kinase-off signaling state, which is characterized by tight association of the PAS and HAMP domains (17, 22, 23). The tip region of the KCD contacts CheA and CheW and affects CheA autophosphorylation as with other chemoreceptors (16, 17, 24, 25).

The PAS domain is comprised of an N-cap (residues 1–19), a PAS core made up of an antiparallel β-sheet and several α-helices (residues 20–119), and a C-terminal helix (residues 119–134) that constitutes roughly the first half of a linker region previously termed F1 (residues 130–161) (26, 27). The PAS domain N-cap has the lowest sequence conservation of any region and is essential for Aer function (26, 28). Previous studies have demonstrated that the N-cap is critical for both PAS domain stability and signal conveyance from the FAD-binding pocket to the HAMP domain, either indirectly through mediating conformational signals or directly through interactions with the HAMP domains (23, 26, 29). Random mutagenesis experiments using an Aer mutant deficient in FAD binding revealed three separate mutations (S28G, A65V, and A99V) that each rescue cofactor binding (22). Furthermore, crosslinking experiments coupled with site-directed mutagenesis demonstrate that transient interdimer PAS–PAS interactions contribute to higher oligomeric states *in vivo*, although these associations depend on temperature and appear transient (26).

Here, we report the structure and biophysical characterization of the Aer PAS domain, making use of the triple variant that combines all three flavin-stabilizing residue substitutions (S28G, A65V, and A99V); hereafter, called, Aer-PAS-GVV. Furthermore, the redox chemistry of PAS-FAD is investigated for different FL and PAS Aer species using a coupled deazaflavin photocycle (30, 31). *In vivo* electron spin resonance (ESR) and size-exclusion chromatography small-angle X-ray scattering (SEC–SAXS) provide insight into the nature of the Aer energy-sensing function. These results have important implications for understanding energy sensing in prokaryotes.

## Results

### ENDOR characterization of Aer WT in cells

When solubilized in detergent or incorporated into nanodiscs, the flavin of *E. coli* Aer was reduced by one electron to the ASQ (FAD_ASQ_) state (17). Most flavoproteins more typically form the neutral protonated semiquinone (FAD_NSQ_) as the one-electron reduced state. Thus, we investigated if FAD_ASQ_ formation was indeed the preferred reduced state *in vivo* by overexpressing Aer in BL-21 cells and recording ^1^H-ENDOR spectra of the whole cells. ENDOR detects protons coupled to the unpaired electron spin on the isoalloxazine ring, and the spectra unambiguously distinguish the FAD_ASQ_ from FAD_NSQ_. Cells wherein Aer expression was not induced showed no appreciable flavin signal. The ENDOR spectrum observed for Aer WT in BL21/DE3 cells was consistent with an ASQ species (Fig. 1, *C* and *D*). Three major proton frequencies were observed in the ENDOR spectrum at 10.4, 8.8, and 7.8 MHz. The peak at 10.4 MHz attributes to the H8a protons; this high value of the H8a indicates the presence of an ASQ radical in the sample (32). The proton frequencies of 8.8 and 7.8 MHz attributes to the H1′ and H6 protons, respectively. Furthermore, smaller hyperfine frequencies between 0 and 3 MHz correspond to the solvent/matrix protons as well as other unresolved protons present on the flavin (Fig. 1*D*) (32, 33).

### Stabilization of Aer PAS domain, characterization, and crystallization

**Figure 2:**
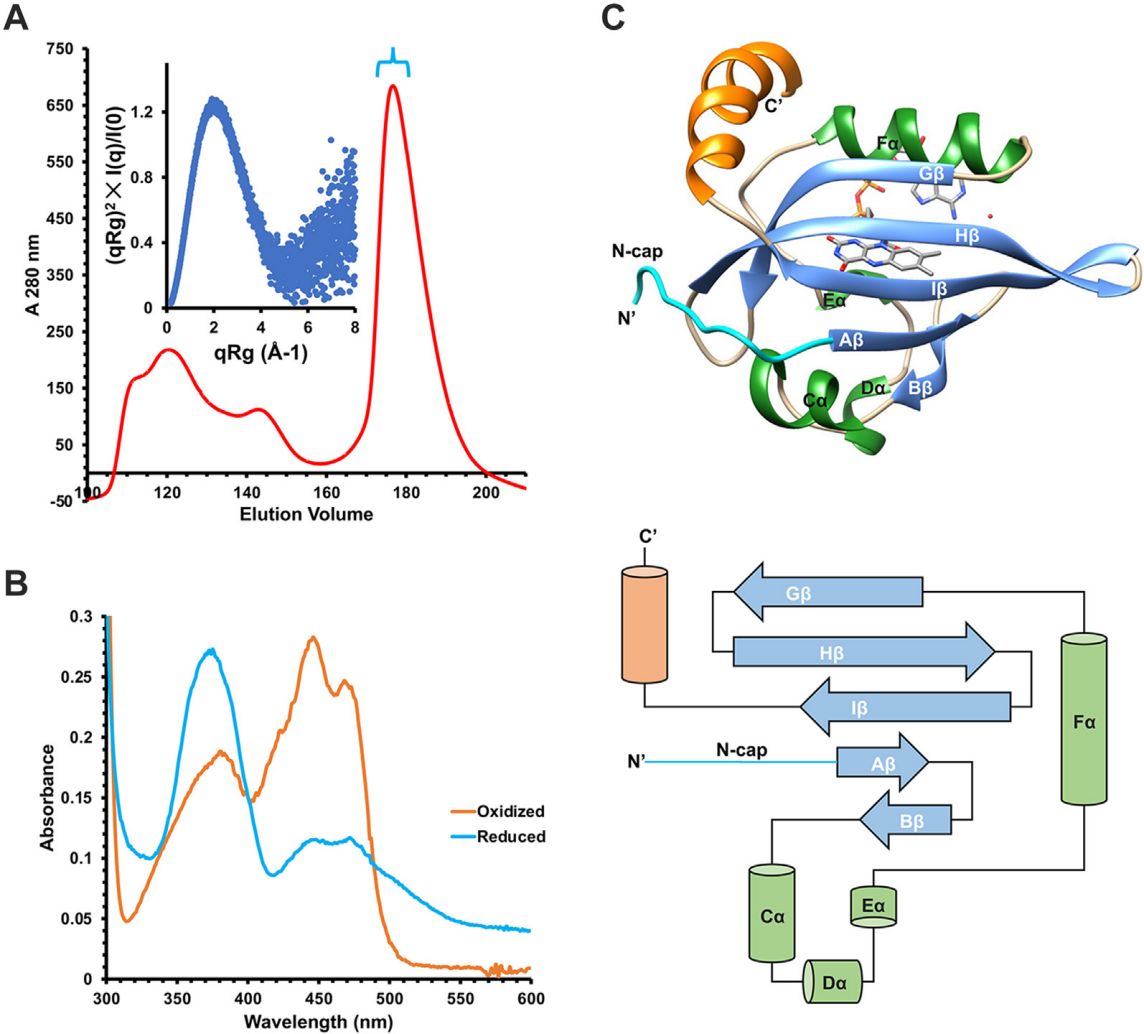
**The properties and structure of Aer-PAS-GVV.***A*, size-exclusion chromatogram of Aer-PAS-GVV on a Superdex S75 preparatory column indicates a well-behaved monomer. *Inset*, SEC–SAXS data of the monomeric Aer-PAS-GVV (indicated by *cyan bracket* on size-exclusion trace). The dimensionless Kratky plot indicates a globular well-folded protein with a step decay in the mid-q region. The calculated molecular weight of Aer-PAS-GVV from the SAXS-derived Porod volume is 19.8 kDa. Higher order aggregates evident on the SAXS elution profile were not further characterized. *B*, oxidized and reduced spectra of the Aer PAS triple mutant Aer-PAS-GVV. The reduced spectrum was achieved using the deazaflavin photocycle. *C*, in the structure of Aer-PAS-GVV, the first 12 residues form the N-cap (*cyan*) and the last 14 form the C-terminal helix (*orange*). Aβ, Bβ, Gβ, Hβ, Iβ, Cα, Dα, Eα, and Fα form the PAS core. Secondary structure schematic of Aer-PAS-GVV depicts a typical PAS domain fold (N-cap and C-terminal helix colored similarly; β-sheets, *light blue*, α-helices, *light green*). PAS, Per–Arnt–Sim; SAXS, small-angle X-ray scattering; SEC, size-exclusion chromatography.

The Aer WT PAS domain was recombinantly expressed in *E. coli* to a minor degree, but it was unstable. Upon purification by SEC, the protein typically eluted as an aggregate bound to GroEL/ES and other chaperones. Watts *et al.* (22) identified three PAS domain residue substitutions (S28G, A65V, and A99V) that each individually rescue FAD binding in FL Aer containing a lesion that abolishes FAD binding. In combination, these three substitutions stabilized the PAS domain to the extent that substantial amounts of Aer-PAS (Aer-PAS-GVV) could be expressed. Aer-PAS-GVV purified as a monomer on SEC, as verified by SEC–SAXS (Fig. 2*A*). The absorbance spectrum of Aer-PAS-GVV showed the characteristic sharpening of the canonical FAD vibrational peaks expected when in a protein binding site (34) (Fig. 2*B*). Like the FL protein, reduction of Aer-PAS-GVV produced the one-electron reduced ASQ (FAD_ASQ_), and further reduction to the HQ form was not possible, even with low-potential reductants such as dithionite (17). Applying multiple stabilizing residue substitutions had cumulative effects on purification yield and on the temperature stability of the PAS domain. For instance, Aer 1-154 S28G must be extracted at 4 °C in buffer containing 20% glycerol to obtain any protein, whereas Aer 1-154 S28G A65V can be purified at room temperature in buffer with as little as 5% glycerol content. Aer-PAS-GVV is even better behaved than the variant with two substitutions. Each of the three substitutions individually have been shown to have minimal impact on signal transduction in Aer and to support chemotaxis in tryptone soft agar (22).

The dimensionless Kratky plot calculated from SEC-coupled SAXS data for Aer-PAS-GVV decays to baseline, consistent with a well-folded globular protein (Fig. 2*A*, *inset*). Although there is prior evidence for PAS–PAS contacts between FL Aer dimers (26), the molecular weight calculated from the SAXS-derived Porod volume for Aer-PAS-GVV (19.8 kDa) is in reasonable agreement with the expected molecular weight of a 14.5 kDa monomer and agrees with the SEC elution profile (Fig. 2*A*).

### Overall structure of Aer-PAS-GVV

The structure of Aer-PAS-GVV was determined to a 2.4 Å resolution by using a molecular replacement probe derived from AlphaFold (35, 36); the structure contains two molecules within the asymmetric unit (Fig. 2*C* and Table 1). The PAS core of Aer-PAS-GVV forms the FAD-binding pocket and consists of five antiparallel β-strands (Aβ, Bβ, Gβ, Hβ, and Iβ) and several peripheral α-helices (Cα, Dα, Eα, and Fα). Flanking the PAS core are an N-terminal coil that extends across a cleft in the β-sheet to the Gβ–Hβ loop and a C-terminal helix that sits above Gβ, Hβ, and Fα. Based upon visual inspection of the protein crystal color, FAD is likely in its oxidized redox state. Discussions of the structure will refer to residue numbering relative to the FL Aer WT sequence.

**Table 1:** Data collection and refinement statistics

Aer-PAS-GVV PDB ID: 8DIK	
Data collection	
Space group	P2_1_
Cell dimensions	
a, b, c (Å)	24.59, 62.95, 78.01
α, β, γ (°)	90, 89.96, 90
Resolution (Å)	49.0–2.40 (2.44–2.40)^a^
*R*_meas_^b^	0.168 (0.586)
I/σI	11.0 (1.6)
Completeness (%)	99.4 (98.7)
Redundancy	6.4 (5.0)
CC_1/2_	0.978 (0.927)
Refinement	
Resolution (Å)	49.0–2.40 (2.53–2.40)
No. of reflections	9349
*R*_work_^c^	0.254 (0.301)
*R*_free_^d^	0.275 (0.353)
No. of atoms^d^	
Protein	1911
Ligand/ion	106
Water	52
*B*-factors (Å^2^)	
Protein	29.1
Ligand/ion	31.2
Water	25.4
Wilson	29.6
R.M.S. deviations	
Bond lengths (Å)	0.003
Bond angles (°)	0.523
MolProbity validation^e^	
MolProbity Ramachandran outliers (%)	0.43
MolProbity Ramachandran favored (%)	95.3
MolProbity rotamer outliers (%)	0.00
MolProbity C-beta outliers	0
MolProbity clashscore	7.8
MolProbity protein geometry score	1.7

a Values in parentheses are for highest resolution shell.

b $R_{\text{meas}}=\frac{\sum_{hl}{\left(\frac{n_{h}}{n_{h}-1}\right)}^{\frac{1}{2}}\left|I_{hl}-I_{h}\right|}{\sum_{hl}I_{hl}}$ QUOTE , where *h* represents the indices of the unique reflections, hl denotes the *n*_*h*_ symmetry–related reflections to *h*, and (|I_hl_-I_h_|) is the absolute difference of the observed intensities to their mean intensity I_h_.

c $R_{\text{work}}=\frac{\sum_{h}\left|\left|F_{o}\right|-\left|F_{c}\right|\right|}{\sum_{h}\left|F_{o}\right|}$where |*F*_o_| represents the observed structure factor and |*F*_c_| represents the calculated structure factor amplitude. R_*f*ree is_ calculated against 10% of the reflections removed from refinement at random.

d Hydrogen atoms not included in counts.

e MolProbity was used to analyze the structure.

Of the two Aer-PAS-GVV molecules in the asymmetric unit; chain B is considerably more disordered and has higher *B*-factors than chain A (Fig. S1*A*). Interestingly, the crystals are pseudo-orthorhombic, with one molecule in the asymmetric unit, but produce much better agreement *R*-factors when refined as monoclinic with two pseudosymmetric molecules of different order. Chain B has a median occupancy-weighted average *B*-factor (OWAB) of 42 Å^2^, twice that of chain A (21 Å^2^), and the first 11 residues of chain B are difficult to resolve in the electron density. Furthermore, the C-terminal α-helix of chain A is much more conformationally homogenous than that of chain B, which retains helicity for five residues only. Real-space electron density fits to chain A are excellent with most residues well defined; whereas, the density quality is lower for chain B (Fig. S1, *B* and *C*). Fewer crystal contacts in the lattice for chain B compared with chain A (1041 Å^2^ of buried surface area *versus* 1335 Å^2^) may produce lower stability and a lessened packing fidelity for chain B. It may also be that partial unfolding or conformational instability of chain B plays a role in nucleation or crystal growth. Further discussion of Aer-PAS-GVV structure refers to chain A, unless otherwise specified.

### Aer-PAS-GVV stabilizing residue substitutions

**Figure 3:**
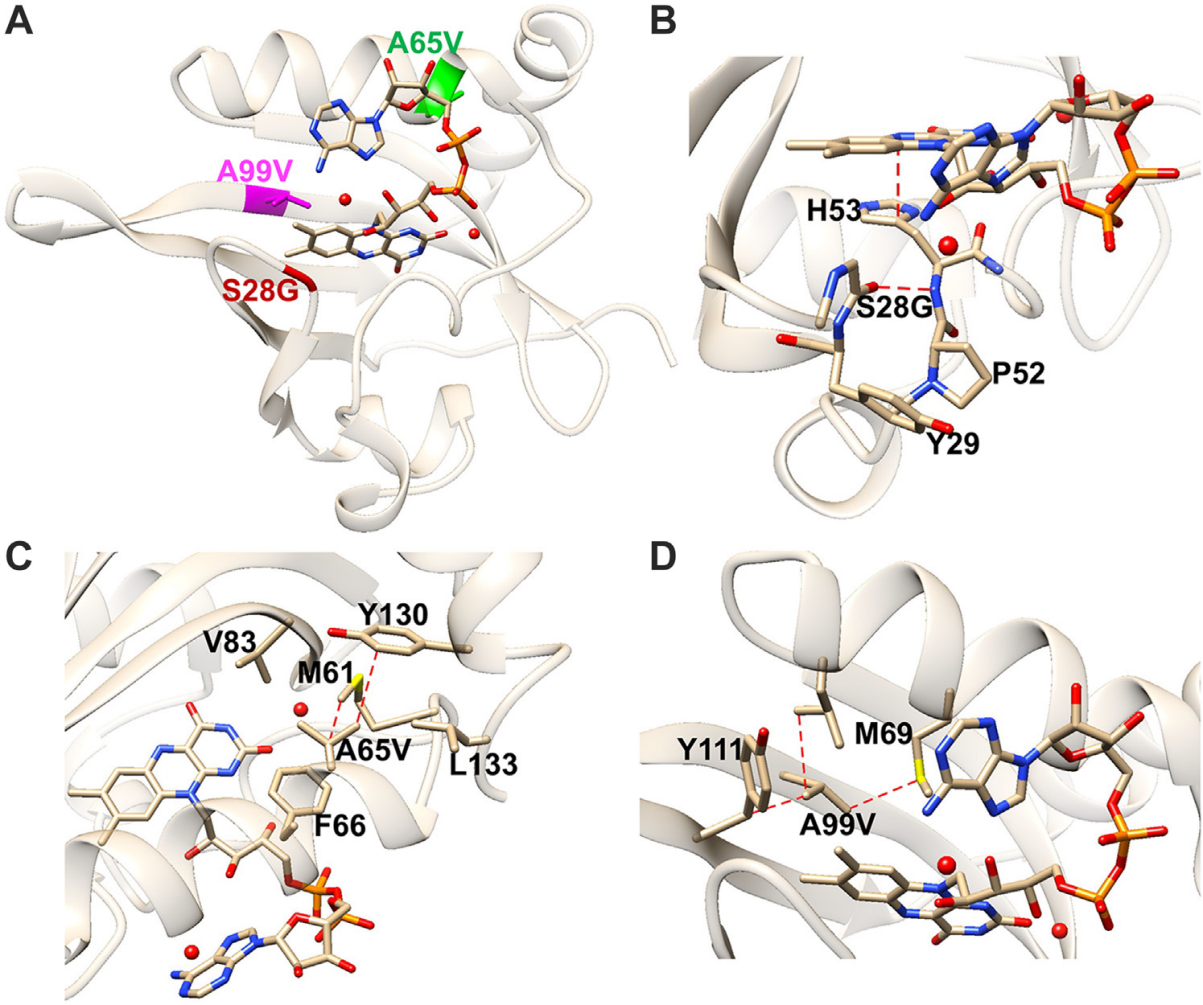
**The three stabilizing residue substitutions of Aer-PAS-GVV.***A*, Aer-PAS-GVV with mutations S28G, A65V, and A99V labeled and colored. *B*, the local environment surrounding G28. *Dotted lines* show interactions mentioned in body text, interacting residues are included for clarity. *C*, the local environment surrounding V65. Residue side chains that form a cavity gating access to flavin-binding pocket are also shown. Hydrophobic interactions with nearby residues are indicated. *D*, the local environment surrounding V99. Valine side chain projects into the binding pocket and interacts hydrophobically with FAD dimethylbenzene ring. Hydrophobic interactions with nearby residues are indicated. PAS, Per–Arnt–Sim.

The three substitutions that stabilize the Aer PAS domain are in relatively close proximity to the flavin (Fig. 3*A*). S28G is on a loop between Aβ and Bβ, with its carbonyl oxygen hydrogen bonding with the amide nitrogen of H53. H53 sits just below the isoalloxazine ring of FAD to which it engages in π–π stacking (Fig. 3*B*). Sequence alignments have previously revealed that a glycine is conserved at position 28 in many other PAS domains, and thus, the S28G substitution may be conducive to flavin binding (28). Modeling a Ser residue into the 28 position does not produce a net increase in favorable interactions for any side-chain rotamer. Although the Ser hydroxyl could form hydrogen bonds with the FAD adenine amine group (as a hydrogen bond acceptor) or with the proximal hydroxyl of the FAD ribose moiety (as either donor or acceptor), either conformation causes steric clashes. Thus, the Ser hydroxyl would unlikely to be accommodated without structural rearrangement in the flavin pocket.

V65 is located at a critical position to stabilize interactions between the C-terminal helix, Gβ, and Fα. The Ala to Val substitution appears to fill in a cavity otherwise produced by M61, Y130, V83, and L133 (Fig. 3*C*). These hydrophobic interactions promote association of the C-terminal helix with Fα and, by extension, with the PAS core. The promotion of flavin binding by A65V indicates that tight association of the C-terminal helix is linked with the stability of FAD in the binding pocket. V65 may be important for maintaining the native fold of the PAS domain in the absence of the HAMP domain, which provides stabilizing interactions with the PAS domain β-sheet in the context of the FL protein (22).

V99 localizes within the PAS core, and its side chain protrudes into the flavin-binding pocket (Fig. 3*D*). The A99V substitution increases hydrophobic interactions to the dimethylbenzene ring of FAD that likely serve to increase cofactor stability in the pocket. The fact that FAD binding and PAS stability are increased by these three non-native substitutions suggests that either interactions with the membrane or the HAMP domain contribute to FAD binding in FL Aer, or that Aer depends upon a loose association with FAD for proper function.

### FAD and the flavin-binding pocket

**Figure 4:**
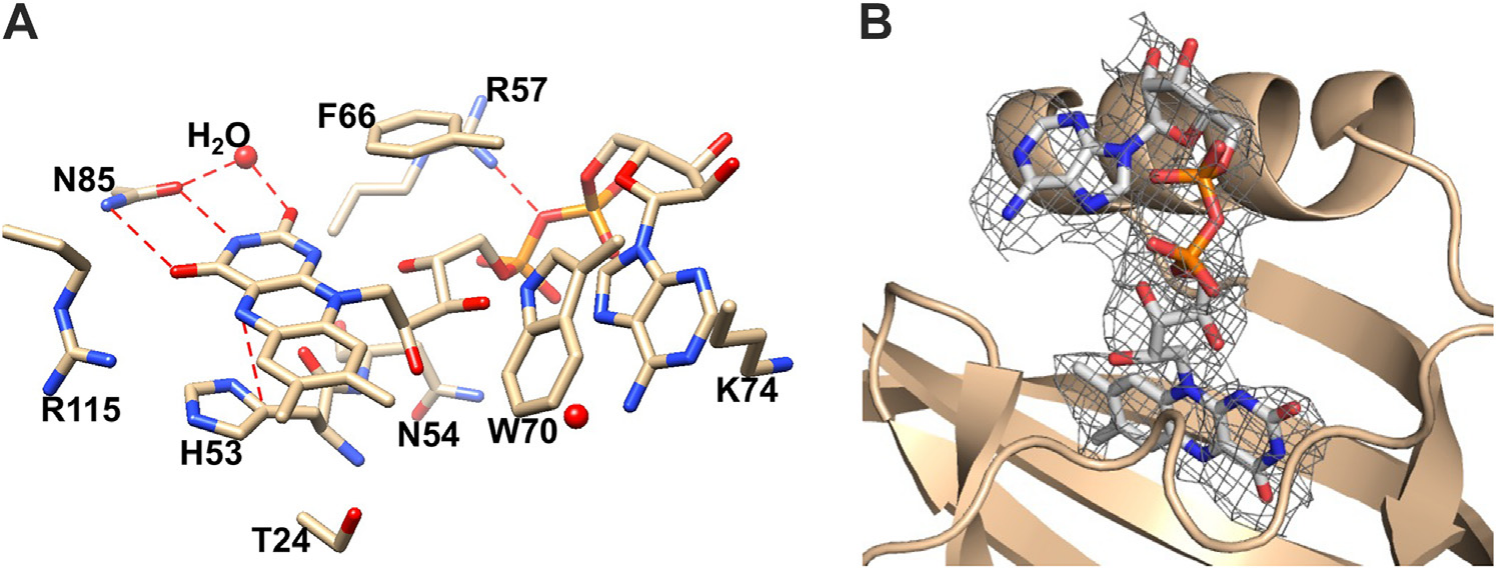
**The Aer PAS FAD-binding pocket.***A*, FAD and nearby residue interactions. Hydrogen bonds and other interactions are demarcated with *dashed lines*. *B*, omit *F*_o_–*F*_c_ map of FAD in binding pocket, contoured at σ = 1.5 (*gray mesh*). FAD, FAD, flavin adenine dinucleotide; PAS, Per–Arnt–Sim.

Residues that contact the bound FAD cofactor of Aer-PAS-GVV reside within the pocket that binds the isoalloxazine ring and extend to peripheral regions to contact the adenosine moiety. The side chain amide nitrogen and carbonyl oxygen of residue N85 hydrogen bonds with the O4 and N3 atoms of the FAD, respectively, and the imidazole ring of H53 forms π–π stacking interactions with the FAD pyrazine ring. A water molecule also hydrogen bonds with the O2 carbonyl of FAD and with the side-chain carbonyl oxygen of N85 (Fig. 4*A*). T27 and M69 project into the flavin-binding pocket and form hydrophobic interactions with the FAD dimethylbenzene ring. R115 is positioned to hydrogen bond with N5, although the distance between the guanidine group and N5 (3.77 Å) exceeds hydrogen bonding distance (Fig. 4, *A* and *B*). Outside the pocket, R57 sits on an irregular coil between helices Eα and Fα and salt bridges with the interphosphate oxygen atom (O3P). W70 π-stacks with the FAD adenine moiety and together with M69 secures the adenine to the peptide scaffold through hydrophobic interactions.

### The Aer N-cap

The primary sequences of PAS domain N-terminal regions are typically poorly conserved; although they often constitute either α-helices or irregular coils. The N-cap of Aer-PAS-GVV forms an irregular coil extending from the N terminus to Aβ at residue L20. Hydrogen bonds between the first few residues of the N-cap (Q9 and N11) with the backbone and side chains of residues on Hβ (H92) appear to stabilize the conformation of the N-cap; interactions with other Aer-PAS-GVV N-terminal residues are minimal until the first residue of Aβ, L20, the backbone of which hydrogen bonds to R115 on Iβ. The R019 side chain projects into the flavin-binding pocket to interact with the isoalloxazine ring of FAD (Fig. 4, *A* and *B*). As previously noted, the N-cap of chain B is not discerned in the electron density, and a lack of stabilizing interactions between its N-terminal region and other residues of Aer-PAS-GVV may account for the conformational heterogeneity of chain B.

### Aer redox potential determination by photoreduction with 5-deazariboflavin

**Figure 5:**
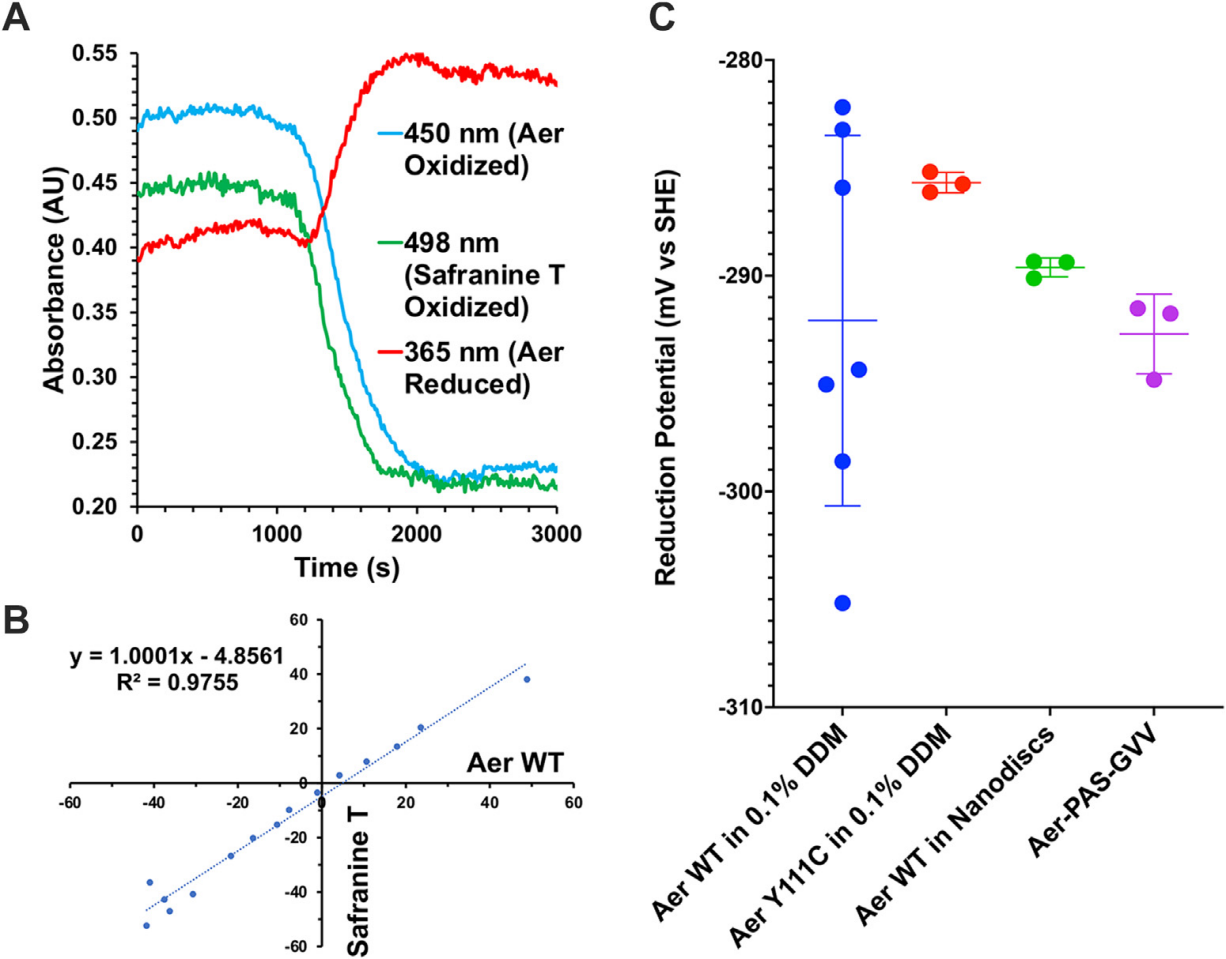
**Aer reduction potential determination using the deazaflavin photoreduction cycle.***A*, kinetic traces of representative wavelengths over the course of reduction. FAD_OX_ absorbs at 450 nm, Safranin T at 498 nm, and FAD_ASQ_ at 365 nm. *B*, representative data of FAD_OX_ and oxidized Safranin T Nernst concentrations plotted against one another in one trial redox titration. The determined redox potential represents one data point in *C*. The intercept specifies the difference between the reduction potentials of FAD and Safranin T. *C*, measured reduction potential values of Aer species reported in Table 2. Averages and standard deviations are indicated. The significances of mean differences were compared with one-way ANOVA: Aer Y111C in 0.1% DDM *versus* Aer WT in nanodiscs (*p* = 0.0004), Aer Y111C in 0.1% DDM *versus* Aer-PAS-GVV (*p* = 0.003), Aer WT in nanodiscs *versus* Aer-PAS-GVV (*p* = 0.047). WT Aer in detergent is not significantly different from the other measurements. DDM, *N*-dodecyl-β-d-maltoside; FAD_ASQ_, FAD anionic semiquinone; FAD_OX_, oxidized FAD.

The reduction potential of FAD bound to *E. coli* Aer was calculated by spectrophotometrically monitoring the redox states of Aer and a redox-sensitive reference dye as both are titrated with a photochemical reducing agent. Upon photoreduction, FAD_ox_ (absorbance at 450 nm) decays to a plateau (Fig. 5, *A* and *B*) as the FAD_ASQ_ (absorbance at 365 nm) grows in. Free FAD when reduced does not accumulate the FAD_ASQ_ state, and therefore, an increase in absorbance at 365 nm concomitant with the decrease in absorbance at 450 nm is diagnostic of bound FAD over the course of reduction. The reduction potentials of the FAD and dye must be sufficiently close to enable the calculation of one from the other, and Safranin T chosen after preliminary experiments demonstrated that it met this requirement for all Aer species tested. Control experiments indicate that the reduction potential measurements are affected drastically neither by temperature changes of 16 °C nor by pH changes of 0.5.

The reduction potentials of Aer-FAD fell between −285 and −292 mV *versus* standard hydrogen electrode (SHE) (Fig. 5*C* and Table 2), which are relatively low reduction potentials for the semiquinone couple of a flavoprotein (37). The reduction potentials of the Aer-PAS-GVV and Aer WT in nanodiscs were similar, −292 ± 2 mV and −289 ± 0.4 mV *versus* SHE, respectively. Whatever the structural changes to the PAS domain, these residue substitutions clearly have little effect on the reduction potential of FAD and therefore should not produce large deviations from the WT PAS fold. The reduction potentials for Aer WT in 0.1% *N*-dodecyl-β-d-maltoside (DDM) were considerably more variable and likely reflect conformational heterogeneity of the PAS domain in detergent given the strong reproducibility of the measurements when the WT protein is either in a lipid bilayer or stabilized by the GVV substitution. Thus, incorporation of the transmembrane domain in a lipid bilayer enhances PAS domain stability, either by an indirect conformational effect or by protecting the PAS domain from detergent interactions. Also noteworthy is the reduction potential calculated for Aer Y111C in 0.1% DDM, at −287.7 ± 0.5 mV *versus* SHE, which is well within the range for Aer WT in 0.1% DDM (Fig. 5*C*). It was previously hypothesized that the Y111C mutation, which swaps signaling outputs (inverted responses), alters the reduction potential of Aer-FAD enough to produce cell tumbling in response to oxygen (13, 26, 38). The results reported here rather indicate that the inverted signaling response produced by Y111C is structural in nature and not because of an alteration of FAD redox chemistry.

**Table 2:** Aer-FAD reduction potentials

Aer species	Reduction potential	n
Aer WT in 0.1% DDM	−292 ± 9 mV	7
Aer Y111C in 0.1% DDM	−285.7 ± 0.5 mV	3
Aer-PAS-GVV	−292 ± 2 mV	3
Aer WT in polar lipid nanodiscs	−289.6 ± 0.4 mV	3

All measurements were taken at 4 °C and pH = 7.0. “n” indicates the number of independent replicates, and errors represent standard deviations, as shown in Figure 5*C*.

## Discussion

Aer is the primary energy sensor for motility in *E. coli* (13, 14). Relationships connecting the structure of the Aer PAS sensory domain, the redox chemistry of its FAD ligand, and changes in cellular respiration are critical factors for understanding *E. coli* energy taxis. The generally accepted model for Aer signaling involves redox-driven changes to FAD that generate a propagation of conformational shifts to the surface of the PAS domain. Interfaces between the PAS and HAMP domain transduce these signals into the coiled-coil domain of the receptor and eventually to the tip region, where they alter the activity of the bound histidine kinase CheA (17, 22, 23, 24, 38). PAS–HAMP associations are uncommon for PAS domains, which tend to function as homodimers or heterodimers (39). Here, we report the crystal structure of a truncated triple mutant of the Aer PAS domain, Aer-PAS-GVV, and characterization of the redox chemistry of PAS-bound FAD using *in vivo* ENDOR and an *in vitro* deazaflavin-based photoreduction system coupled with dye equilibration. The observed redox states of FAD, the relatively low redox potential for the FAD_OX_–FAD_ASQ_ couple, and an uncommon network of isoalloxazine interactions within the binding pocket place constraints on the Aer signaling scheme.

### Comparisons to related PAS sensors provide insight into conformational signaling

**Figure 6:**
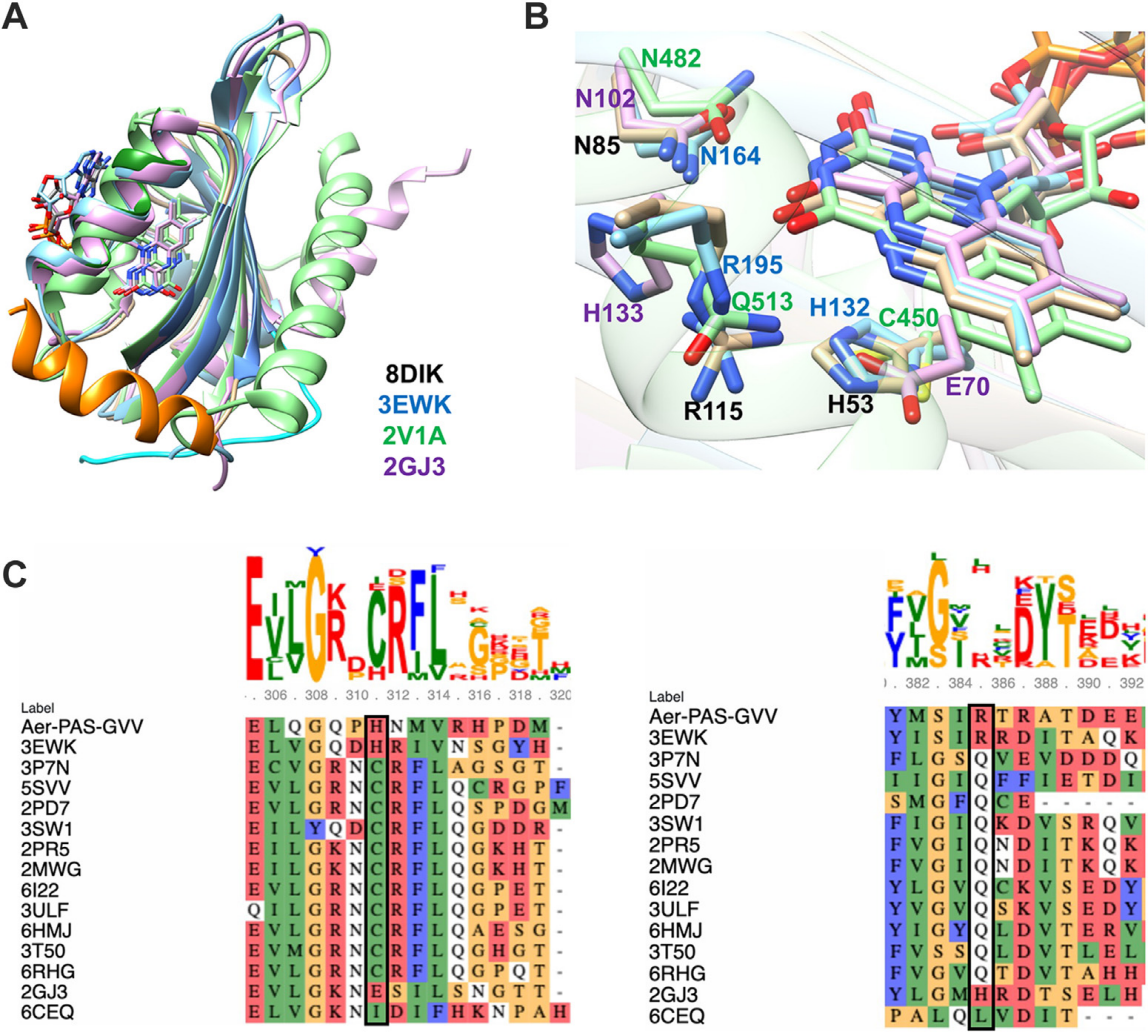
**Structural comparison of Aer-PAS-GVV to other PAS domains.***A*, superposition of Aer-PAS-GVV (Protein Data Bank [PDB] ID: 8DIK, colored *tan* with *cyan* N-cap and *orange* C-terminal helix), with the PAS domains of *Methylococcus capsulatus* MmoS (PDB ID: 3EWK, *blue*), *Azotobacter vinelandii* NifL (PDB ID: 2GJ3, *purple*), and the LOV2 domain of *Avena sativa* phototropin 1 (PDB ID: 2V1A, *green*). *B*, close up of the flavin-binding pockets of the PAS domain superposition shown in (*A*). Residues superimposing with Aer-PAS-GVV residues H53, N85, and R115 (labeled in *black*) are displayed and colored according to PDB ID as in (*A*). *C*, sequence alignments of Aer-PAS-GVV with other FAD and FMN-binding PAS domains. On the *left* is the alignment in the region around Aer-PAS-GVV residue H53, marked with a *black rectangle*. On the *right* is the alignment in the region around Aer-PAS-GVV residue R115, marked with a *black rectangle*. Sequence conservation logos are shown above. FAD, flavin adenine dinucleotide; LOV, light–oxygen–voltage; PAS, Per–Arnt–Sim.

In order to propagate a redox signal, the Aer PAS domain must undergo a conformational change in response to the FAD_OX_–FAD_ASQ_ transition. Comparisons to the structure and properties of related flavin protein redox sensors give some indications as to what such conformational transitions may involve. Overall, the interactions formed within the FAD-binding pocket are analogous to the extended hydrogen-bond network found in PAS-A of MmoS, another putative *E. coli* redox sensor (Fig. 6, *A* and *B* and Video S1) (40). MmoS binds FAD and is the sensory module of a two-component signaling system regulating soluble methane monooxygenase (40, 41). MmoS contains corollary residues to Aer R115, N85, H53, and T18 (R195, N164, H132, and T103, respectively) that produce similar interactions to those observed in *E. coli* Aer PAS. MmoS residue 107, which is a native glycine, corresponds to one of the Aer-PAS-GVV stabilizing substitutions, G28. Intriguingly, the reduction potential of MmoS–FAD compares closely to that of Aer-FAD at −290 ± 2 mV *versus* SHE, suggesting that these common residues and their hydrogen bonds, van der Waals, and electrostatic interactions with flavin are critical determinants for the FAD redox chemistry observed in both cases (41). H53 and R115 are flavin pocket residues somewhat uncommon for flavin-binding PAS domains (Fig. 6*C*) (42, 43, 44). Equivalent residues between *E. coli* Aer (H53 and R115) and *E. coli* MmoS (H132 and R195) suggest corresponding roles and may reflect a common signaling mechanism (Fig. 6*B*). Positively charged side-chain residues such as histidine and arginine in the flavin microenvironment may tune the flavin reduction potential by providing stabilizing electrostatic interactions for additional electron density at the isoalloxazine ring upon reduction (45).

In PAS domains, interactions between the flavin and the protein at the Aer 53 position coordinate protein conformational responses with changes in cofactor chemistry. For example, the photocycle of light–oxygen–voltage (LOV) signaling domains depends on a reactive cysteine residue at the 53 position of Aer. H53 aligns with *As*LOV2 C450, which forms a covalent adduct with the C4a position of *As*LOV2 FMN upon flavin excitation (Fig. 6*B*) (46). When the Aer flavin reduces from FAD_OX_ to FAD_ASQ_, the H53 side chain may change conformation or protonation state. Notably, there are no obvious hydrogen-bond donors or acceptors in the vicinity of H53 and thus, if it were to become positively charged, a drive to satisfy hydrogen-bonding partners may elicit conformational changes in the flavin pocket, similar to mechanisms characterized for light-sensing cryptochromes (47, 48, 49).

R115 also resides in close proximity to the flavin and likely modulates its reactivity. R115 does not form a strong interaction with N5 of the isoalloxazine ring but may function to keep solvent from interacting with bound FAD and thereby protect against conversion to the NSQ (FAD_NSQ_). Interactions between R115 and FAD may increase upon conversion of the flavin to the FAD_ASQ_, but the relatively high p*K*a would prevent N5 protonation (50). Consistent with Aer R115 (and its analog R195 in MmoS) acting to stabilize the low reduction potentials of their flavin cofactors; substitution of R237 to alanine in *Methylophilus methylotrophus* electron-transferring flavoprotein (*Mm*ETF) increases the FAD reduction potential by ∼200 mV (51). Similar to Aer and MmoS, the guanidium moiety of R237 sits ∼3.7 Å above the isoalloxazine N5 in the *Mm*ETF crystal structure where it is thought to stabilize the ASQ FAD reduction product by providing a kinetic block to protonation and full reduction of the flavin (51, 52). If the Aer-FAD_ASQ_ state promotes a stronger interaction between R115 and the isoalloxazine N5, movement of either the FAD molecule or Iβ, or both, could result. Such movements could be important for the conformation of the β-sheet, which interacts with the HAMP domain and is essential for signal transmission (23). It is also possible that residue R115 forms new interactions with other residues as part of the conformational shifts involved in switching signaling states, as with the corollary residue Gln residues of photosensory LOV domains (46, 53, 54). The R115 main chain hydrogen bonds across the β-sheet to L20, the first Aβ residue, and first residue C-terminal to the N-cap. Conformational shifts important for signal conveyance elicited by R115 interactions with FAD may therefore involve the N-cap and N-terminal Aβ residues.

N85 may also play a role in conformational signaling. The N85 side chain hydrogen bonds to FAD N3 and O4, whereas the N85 peptide backbone participates in a network of interactions proximal to the anchor points for the N-cap. Conformational changes in N85 in response to flavin reduction may increase interactions between the N85 side chain and the FAD uracil ring, events that would propagate through the β-sheet and alter hydrogen bonds that hold the N-cap to the PAS core. One of the functions of the N-cap may be to stabilize the PAS domain fold (26). However, evidence that the N-cap is important for signal conveyance and not simply proper folding of the PAS domain (23, 26, 29) suggests that the N-cap adopts different orientations in different signaling states and may even release from the PAS domain core. A released N-cap would provide entropic stabilization (55) and may alter the structure of the PAS domain near L20 and Aβ.

### Aer energy sensing

It is largely unknown what cellular factors communicate with Aer; however, the structural and redox properties of AerPAS put constraints on what those partners may be. As Aer monitors the metabolic load of the ETC, it must be sensitive to either a respiratory complex or some other factor with a reduction potential low enough to reduce FAD_OX_ to FAD_ASQ_. Several respiratory chains in *E. coli* contain oxidoreductases that meet the requirements for the Aer redox partner. The standard reduction potential of the redox couple pair NAD^+^/NADH is roughly −320 mV *versus* SHE. Similarly, H^+^/H_2_ has a standard potential of −420 mV and HCO_3_^−^/HCO_2_^−^ has a standard potential of −430 mV (56). The reduction potentials of NADH dehydrogenases I and II (genes *nuoA-N* and *ndh*), hydrogenases I and II (genes *hyaA-F* and *hybA-G*), and the two formate dehydrogenases (genes *fdnGHI* and *fdnGHI*, *fdhf*, *hycA-H*) generate reducing equivalents of potential low enough to donate electrons to Aer-FAD, and some combination of these enzymes or their products may be responsible for Aer sensitivity to the ETC. Sensing behavior of electron transport mutants that produce varying H^+^/e^−^ ratios suggests that Aer signaling is coupled to NADH dehydrogenase I (15). However, energy taxis in the absence of NADH dehydrogenase I (15, 56) may derive from donor redundancy and perhaps involvement of the hydrogenases or formate dehydrogenases.

Complex II (succinate dehydrogenase, Sdh), ubiquinone, menaquinone, and NADH-dependent flavin oxidoreductase (FRE) have also been discussed as possible Aer redox partners (28). Aer directs motility under conditions where succinate is the primary carbon source, despite the standard reduction potential of the succinate/fumarate redox couple being only 30 mV (56, 57), much higher than the Aer flavin potential of −290 mV. Thus, Sdh cannot be a direct electron donor to Aer-FAD_OX_; neither can be ubiquinone or menaquinone, whose potentials are also too high to produce the Aer-FAD_ASQ_. Under growth on succinate, Aer is likely reduced by an NAD(P)H coupled system; NADH being produced by C_4_-dicarboxylic acid metabolism of succinate in the tricarboxylic acid cycle, for example. The FRE has also been suggested as a possible Aer redox partner (13). Although the FRE flavin potential is likely appropriate to reduce Aer, FRE typically reduces free flavin to the HQ, FADH^−^ state, which does not appear stable in Aer (58). Although their formal potentials are too high to be Aer reductants, Sdh and quinones could act as Aer oxidants to generate smooth swimming states when terminal electron acceptors are introduced.

**Figure 7:**
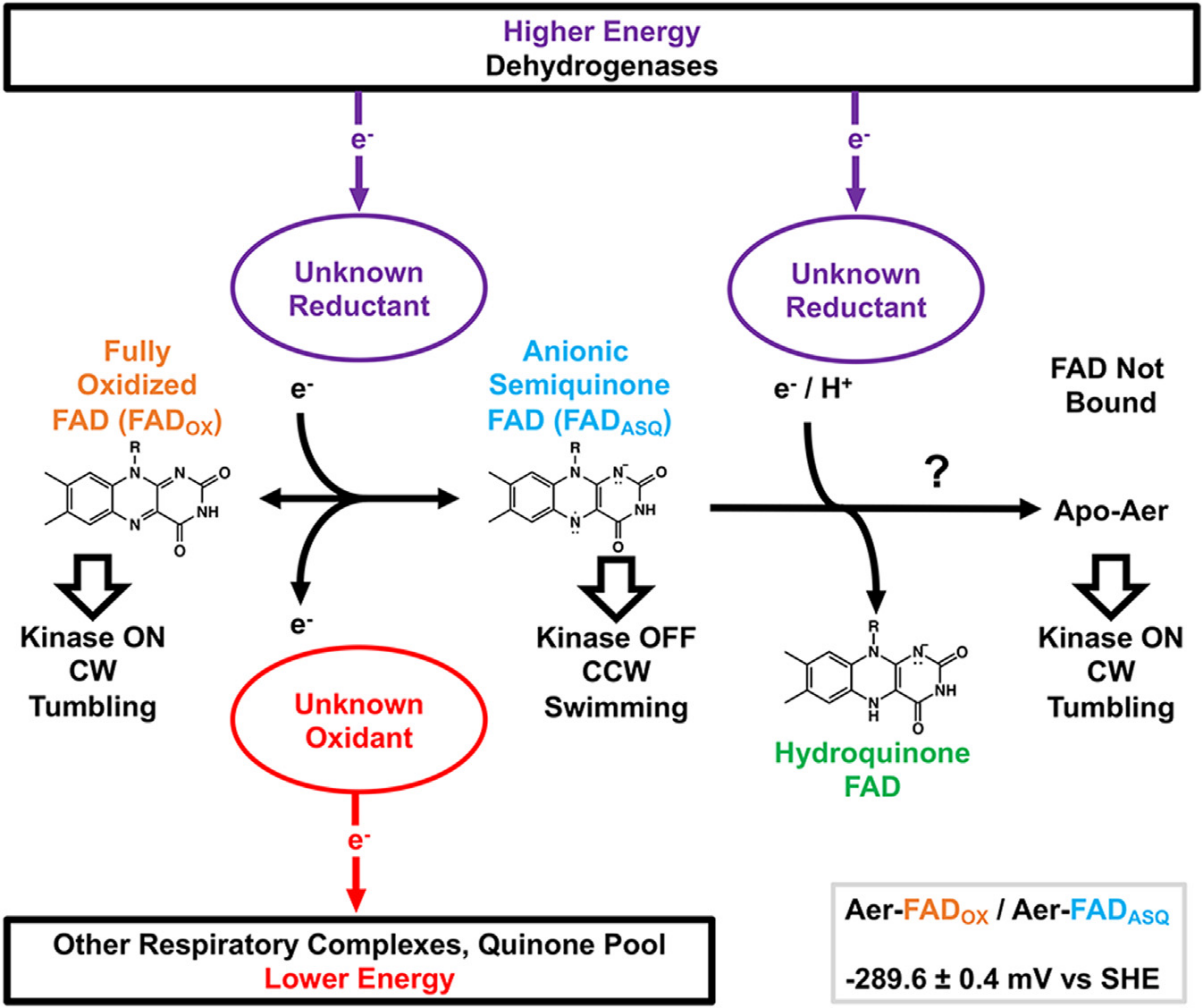
**Aer energy sensing model.** FAD bound to the Aer PAS domain has a reduction potential of −289.6 ± 0.4 mV *versus* SHE, as determined for nanodisc-reconstituted Aer WT. An unknown higher energy reductant reduces FAD_OX_ to FAD_ASQ_, switching the Aer signaling state from kinase ON to kinase OFF. Aer-FAD_ASQ_ then reduces an unknown oxidant, switching the signaling state back. Thus, Aer signaling events are determined by the availability of both higher energy reductant species and lower energy oxidant species, conferring sensitivity to the flow of reducing equivalents within respiratory chains at *higher* and *lower* energies, “upstream” and “downstream.” FAD, flavin adenine dinucleotide; FAD_ASQ_, FAD anionic semiquinone; FAD_OX_, oxidized FAD; PAS, Per–Arnt–Sim; SHE, standard hydrogen electrode.

Cell taxis requires switching of the flagellar rotation direction, modulated by receptors altering their signaling states (5). Two redox states have now been characterized between which Aer-FAD can switch: FAD_OX_ and FAD_ASQ_ (Fig. 7). However, swimming behavior of *E. coli* under different nutrient environments indicate that a third redox state of Aer participates in the signaling mechanism. Aer causes *E. coli* to switch to smooth swimming (kinase-off) when cells are either (1) supplied with an electron donor after having previously obtained a fully oxidized respiratory system or (2) supplied with an electron acceptor (*e.g.*, oxygen) after having previously obtained a fully reduced respiratory system (38). The latter condition implies that a form of Aer present under highly reducing conditions is oxidized to the Aer-FAD_ASQ_ state to cause smooth swimming (kinase off). Reduction of Aer-FAD to a kinase-on HQ state (Aer-FAD_HQ_) under a highly reducing environment (*e.g.*, anaerobiosis), with subsequent oxidation to Aer-FAD_ASQ_ upon introduction of electron acceptors would match the physiological data (13). However, Aer-FAD_HQ_ has not been observed. In nanodiscs, Aer-FAD undergoes an FAD/FAD^•–^ redox couple at relatively low potential (−290 mV). Conversion to the fully reduced HQ state FAD^•−^/FADH^–^ requires further protonation of the FAD_ASQ_ to the NSQ (FAD_NSQ_, FADH^•^), which is not favorable for purified Aer at physiological pH. The cellular ENDOR results indicate that the FAD^•–^ state is also accessed in cells, with little evidence for formation of FAD_NSQ_. Thus, unless the specific environment of Aer at natural expression levels alters the flavin reactivity, FAD/FAD^•–^ is the primary redox couple of Aer. It is possible that the binding affinity of the Aer PAS domain for FAD_HQ_ is so low that FAD_HQ_ is released from the protein, although Aer-FAD_ASQ_ accumulates *in vitro*, even with high potential reductants (Fig. 5*A*). Perhaps a critical structural determinant for achieving Aer-FAD_HQ_ is not present; factors affecting the conformation of the PAS domain and the flavin microenvironment could include unidentified protein-binding partners, signals received through PAS–membrane interaction, or even covalent modification of Aer. A kinetic or thermodynamic block may exist that prevents the formation of Aer-FAD_HQ_, a block that may be removed *in vivo* by an unidentified factor. Altering the conformation of R115 to modify electrostatic interactions stabilizing the Aer-FAD_ASQ_ state may provide a basis for selective formation of Aer-FAD_HQ_, much as substituting R237 for alanine in *Mm*ETF facilitates formation of FAD_HQ_ (51).

We are left with a conundrum in that Aer signaling is unlikely to be two state, yet, the fully reduced FAD_HQ_ state does not appear to be stable in the isolated protein. Thus, under a fully reduced ETC, either the Aer cellular environment stabilizes the FAD_HQ_ state, or Aer becomes uncoupled from the ETC. One possibility is that when the flavin pool becomes fully reduced, FADH^−^ does not bind Aer and the PAS domain absent from flavin then generates a kinase-on state (Fig. 7). In support of this view, many mutation sites that map to the flavin pocket produce kinase-on states and the Aer-PAS-GVV variant, which stabilizes flavin binding, favors kinase-off (29). This scheme would still generate the required multistate signaling behavior dependent on three redox states of FAD; however, the FAD_HQ_ state may not be Aer bound.

## Experimental procedures

### Chemicals, protein overexpression, and purification

All proteins used in this study were derived from and produced in *E. coli*. Membrane-scaffold protein D1 (MSP1D1), Aer, and all Aer variants were recombinantly expressed in BL21/DE3 *E. coli* cells using a kanamycin-resistant pET28a vector and purified by affinity chromatography with a genetically encoded N-terminal 6xHis tag with thrombin cleavage site. 5-Deazariboflavin was purchased from Santa Cruz Biotechnology; IPTG and high-density cobalt agarose beads were purchased from GoldBio; FAD was purchased from Chem-Impex International; and DDM was purchased from Anatrace. Tris buffer, HEPES buffer, KCl, NaCl, and MgCl_2_ were purchased from Sigma.

All proteins were transformed into BL21/DE3 cells and plated on LB agar containing 50 μg/ml kanamycin. A single colony was selected and grown in a 50 ml Luria broth overnight, which was then used to inoculate 8 L of autoclaved Terrific broth. Protein expression was induced by addition of IPTG to 1 mM at an absorbance of 0.6 to 0.8 at 600 nm following growth at 37 °C. Following induction, the culture was incubated shaking at 17 °C for an additional 16 to 20 h. Cells were pelleted by centrifugation and frozen. Reduction of temperature was not used for growing the membrane scaffold protein MSP1D1, and induction was stopped 3 h after IPTG addition to reduce proteolytic degradation and purified as previously described (59, 60).

Purification of Aer WT and its FL variants involved solubilization of a frozen 8 L cell pellet in chilled (4 °C) 50 mM Tris (pH 8.0), 500 mM KCl, 10% glycerol (extraction buffer), and sonication for 6 min. Ice was packed around the sonicated solution to reduce the risk of temperature increase. The membrane fractions were harvested by centrifugation at 12,096*g* and resuspended in chilled extraction buffer containing 1% DDM and supplemented with 1 mM PMSF and 10 μg/ml FAD. Solutions were inverted continually at 4 °C for 5 to 5.5 h and then centrifuged at 48,384*g* for 1 h. The supernatant was extracted and incubated with 5 to 10 ml of cobalt resin for 12 to 16 h at 4 °C, then washed with 25 mM Tris (pH 8.0), 150 mM NaCl, 10% glycerol, 0.1% DDM (wash buffer) supplemented with 10 μg/ml FAD and eluted with 25 mM Tris, 150 mM NaCl, 300 mM imidazole, 10% glycerol, 0.1% DDM (pH 8.0) (elution buffer) at 4 °C. Aliquots were flash frozen and stored at −80 °C. The PAS domain construct (Aer 7–135 S28G A65V A99V, the Aer PAS triple mutant, or Aer-PAS-GVV) was purified at room temperature following sonication for 6 min on ice. The supernatant was clarified by centrifugation at 20,000 rpm and loaded onto nickel–nitrilotriacetic acid resin and then eluted with elution buffer. The Aer-PAS-GVV domain was enriched by size-exclusion purification on an S75 preparatory column. Relevant fractions were pooled and buffer exchanged into 25 mM Tris (pH 8.0), 150 mM NaCl, and 20% glycerol. Aer-PAS-GVV used in crystallization was incubated overnight with thrombin to cleave the 6xHis tag and enriched by size-exclusion purification on an S75 analytical column. Aliquots of protein were flash frozen and stored at −80 °C. Protein concentration was calculated using an FAD standard curve by measuring the sample flavin absorbance at 450 nm.

### Crystallization of Aer-PAS-GVV, data collection, structure determination, and refinement

Initial crystals of Aer-PAS-GVV were produced from a commercial screen (Hampton) using a 96-well sitting drop tray. Optimization of pH, salt identity, salt concentration, glycerol content, and precipitant was then performed using hanging drop vapor diffusion. Crystallization was accompanied by large amounts of precipitation, making crystals difficult to identify and isolate. Multiple needle-shaped overlapping single crystals grew from nucleation sites, which posed significant technical challenges to selecting single crystals for diffraction. Crystal formation required several days of incubation, and crystal quality greatly improved upon streak seeding. For diffraction quality crystals, aliquots of Aer-PAS-GVV were concentrated to 20 mg/ml in 25 mM Tris (pH 8.0), 150 mM NaCl, 20% glycerol, and crystallized by hanging drop vapor diffusion (1 μl protein, 1 μl crystallization solution) at 4 °C in 50% (w/v) PEG-3000, 100 mM Tris (pH 7.0), 150 mM NaCl, and 20% glycerol. No exogenous reductants were added, and crystals were therefore formed under oxidized conditions. X-ray diffraction data were collected on Aer-PAS-GVV crystals at 0.968 Å on the CHESS F1 beamline equipped with a DECTRIS EIGER X 16M pixel array detector. Rapid cooling in liquid nitrogen and storage at cryogenic temperatures was challenging given the size and fragility of the crystals; instead, protein crystals were cryoprotected in mother liquor and frozen in a cryostream prior to X-ray exposure.

Data reduction and scaling were performed with HKL-2000 (HKL Research Inc) (61). Data refinement and phasing were performed in PHENIX (62). The structure of Aer-PAS-GVV was determined by molecular replacement in PHENIX using an AlphaFold prediction (35, 36) for Aer 7–134 S28G A65V A99V, supplemented with FAD coordinates from the N-terminal PAS domain of NifL (Protein Data Bank ID: 2GJ3). Multiple rounds of model building were performed in COOT and PHENIX, during which water molecules were added (62, 63). The Aer structure refined best in a monoclinic space group with two molecules per asymmetric unit, despite the crystal form being pseudo-orthorhombic with one molecule per asymmetric unit. However, refinement in orthorhombic produced much high *R*-factors. Although pseudosymmetry relates the two monoclinic molecules, one appears much less ordered than the other (Fig. S1). The coordinates and structure factors for Aer-PAS-GVV have been uploaded to the Protein Data Bank as entry 8DIK. Data processing and refinement statistics are listed in Table 1.

### Nanodisc reconstitution of FL Aer

Following purification of Aer WT or its variants in 1% DDM, the protein was concentrated to as high a degree as possible (∼20 mg/ml). Stoichiometric ratios of 1:5:50 Aer:MSP1D1:lipids were calculated for a final sample volume of 500 μl. All ingredients were combined on ice. To begin, a solution of 20 mM sodium cholate and lipids was prepared and incubated for 15 min. At this stage, glycerol was added from an 80% glycerol stock solution to the sample to maintain a final concentration of at least 10%. Aer was then added and incubated for 15 min before addition of MSP1D1. The sample was incubated rocking at 4 °C for 45 min, after which 400 μl of hydrated BioBeads were added. Incubation at 4 °C proceeds for another 60 min, at which time the liquid sample is extracted and filtered through a 20 μm filter and injected onto a Superose 6 analytical column pre-equilibrated with 25 mM Tris (pH 8.0), 150 mM NaCl, and 10% glycerol. Fractions containing Aer at one dimer per disc (as determined by 450 nm absorbance and elution position) were pooled and concentrated (17, 59).

### Deazaflavin photochemical reduction and spectrophotometric measurement of the Aer reduction potential

Aer WT and its variants, either FL or Aer-PAS-GVV, were buffer exchanged into 20 mM Na_3_PO_4_, 15 mM EDTA, 150 mM NaCl, 20% glycerol, pH 7.0 in preparation for chemical reduction *via* the 5-deazariobflavin photocycle (31). The solution was loaded into a quartz cuvette and exposed to a mixed nitrogen (97%) and hydrogen (3%) atmosphere in an anaerobic chamber to remove oxygen in the gas above the cuvette solution, 5-deazariboflavin was added to a final concentration of 5 μM from a stock solution of 5-deazariboflavin dissolved in dimethyl sulfoxide, then the cuvette was sealed with a transparent quartz cap, and wrapped with parafilm before being removed from the anaerobic chamber. An Agilent UV–vis spectrometer was used to monitor species in solution and blanked with the starting solution. The spectrometer cell was kept at a constant 4 °C over the course of the experiment with the use of a Quantum Northwest TC125 temperature controller. The 5-deazariboflavin photocycle was initiated with 300 nm light provided by a lamp positioned over the transparent quartz cap. In cases where reduction potential measurements were performed, a redox sensitive dye, in this case Safranin T, was added to the cuvette while in the glove bag. The amount of Safranin T added was selected to produce an absorbance band at 498 nm of comparable intensity to the absorbance band of bound FAD at 450 nm. The absorbance band of fully oxidized flavin (FAD_OX_) at 450 nm, the absorbance band of ASQ (FAD_ASQ_) at 365 nm, and the absorbance band of oxidized Safranin T at 498 nm were monitored over the course of the experiment. Characteristic wavelength traces begin with a plateau near zero and then change gradually in the manner of a sigmoid curve. Data collection was ended once the absorbance bands reach their second plateaus, signifying full reduction of the species in question.

The reduction potential of FAD bound to Aer and its variants is calculated using the known reduction potential of the reference dye as previously described (30). As electrochemical potentials are equal at equilibrium:

(1) $$E_{x}=E_{m,x}+\frac{RT}{nF}\ln\left(\frac{x_{ox}}{x_{red}}\right)$$

(2) $$E_{m,D}+\frac{RT}{nF}\ln\left(\frac{D_{ox}}{D_{red}}\right)=E_{m,P}+\frac{RT}{nF}\ln\left(\frac{P_{ox}}{P_{red}}\right)$$

where “x” connotes a given chemical species, “D” connotes dye, and “P” connotes protein. Here, R is the universal gas constant, F is Faraday's constant, T is the temperature in Kelvin, and n is the number of electrons involved in the reduction; for both protein and dye, n = 1. The relative concentrations of the oxidized and reduced forms of each chemical species (dye and protein) are calculated using absorbance minima and maxima for every time point in the kinetic traces:

(3) $$\frac{A-A_{\min}}{A_{\max}-A}=\frac{\left[oxidized\right]}{\left[reduced\right]}$$

The ratios of the oxidized and reduced forms of each species are then used to calculate their Nernst concentrations:

(4) $$y=\frac{RT}{nF}\ln\left(\frac{D_{ox}}{D_{red}}\right)$$

(5) $$x=\frac{RT}{nF}\ln\left(\frac{P_{ox}}{P_{red}}\right)$$

These values are then plotted against one another and fit to a linear regression in Microsoft Excel:

(6) $$y=\Delta E_{m}+x$$

From this linear regression, the difference between known dye reduction potential and the unknown FAD reduction potential can be calculated:

(7) $$\Delta E_{m}=E_{m,P}-E_{m,D}$$

Diagnostics were inspected to verify successful reduction potential measurements. First, concomitant and proportional increase in 365 nm (FAD_ASQ_) with reduction in 450 nm (FAD_OX_) indicated that over the course of reduction, FAD remained bound to protein, as FAD_ASQ_ did not form to spectrophotometrically significant levels when FAD is free in solution. Second, plateaus in kinetic traces with slopes near zero indicated complete reduction. Third, FAD_OX_ (450 nm) and oxidized Safranin T (498 nm) reduction began and concluded at very nearly the same times, indicating that the two species have reduction potentials that lie close enough together for reliable calculation of the reduction potential of FAD. Finally, a Nernst plot with a slope near 1 (Equation 6) indicates that the protein or dye was not undergoing other reactions that might complicate data analysis. Datasets yielding slopes between 0.88 and 1.22 were selected.

### SEC–SAXS

SAXS data were collected at CHESS G1 line on the Finger Lakes CCD detector. About 100 μl of Aer 7–134 S28G A65V A99V sample containing 4.5 to 5.5 mg/ml protein was injected onto a Superdex 75 Increase Small-Scale Analytical Column pre-equilibrated with sample buffer (50 mM HEPES [pH 8.0], 150 mM KCl, and 10 mM MgCl_2_) coupled to the G1 SAXS sample cell. The monodisperse stream was fed through a Wyatt MALS/DLS and dRI detector, and data were collected with 2 s exposure times to the X-ray beam at a flow rate of roughly 0.2 ml/min. RAW was used to process the SEC–SAXS data and to generate a Guinier Fit and dimensionless Kratky Plot (64, 65).

### ESR spectroscopy measurements in cells

Aer WT and Aer E483Q were expressed with 1 mM IPTG overnight at 4 °C in BL21/DE3 cells using a pET28a vector and pelleted by centrifugation. Cells were then resuspended in a 25 mM Tris (pH 8.0), 150 mM NaCl, and 25% glycerol solution and then loaded into an ESR capillary. The cells were then pelleted a second time within the tube by centrifugation, and ESR measurements were taken. Pulse ENDOR measurements were carried out on a Bruker E580 spectrometer operating at Q-band (∼34 GHz) equipped with a 10 W solid-state amplifier, 150 W RF amplifier, and an AWG. The Davies sequence (*π*-*t*_1_-rf pulse-*t*_2_-*π*/2*-t*-*π*-*t*_echo_) was used to carry out the ENDOR measurements. A selective microwave pulse of around 74 (*π*/2) and 150 ns (*π*) was used along with a 20 μs radiofrequency pulse. The resonator was critically coupled, and the measurements were carried out at 150 K.

## Data availability

The coordinates and structure factors for Aer-PAS-GVV have been deposited in the Protein Data Bank as entry 8DIK. All other data are included within this article or available in the online supporting information.

## Supporting information

This article contains supporting information (Fig. S1 and Video S1).

## Conflict of interest

The authors declare that they have no conflicts of interest with the contents of this article.
