# Supplementary material for: Redox properties and PAS domain structure of the Escherichia coli energy sensor Aer indicate a multistate sensing mechanism
Source: J Biol Chem. 2022 Oct 15;298(12):102598. doi: 10.1016/j.jbc.2022.102598 (PMC9668731; doi:10.1016/j.jbc.2022.102598)
Supplement: Supplemental information [file mmc1.docx]

**Supporting Information for:**

**Interdomain linkers regulate histidine kinase activity by controlling subunit interactions**

Zachary Maschmann, Siddarth Chandrasekaran, Teck Khiang Chua, and Brian R. Crane*

Department of Chemistry and Chemical Biology, Cornell University, Ithaca, NY 14850

*For Correspondence: bc69@cornell.edu

The PDF file includes:

**Supplemental Figure 1** – Comparison of the two AerPAS-GVV molecules within the asymmetric unit in terms of B-factors and electron density values.

**Supplemental Video 1** – Video showing the superpositions of Figure 6.

**
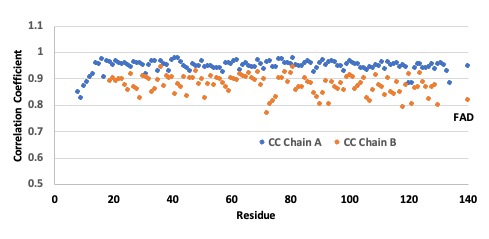
**

**B**

**C**

**A**

**
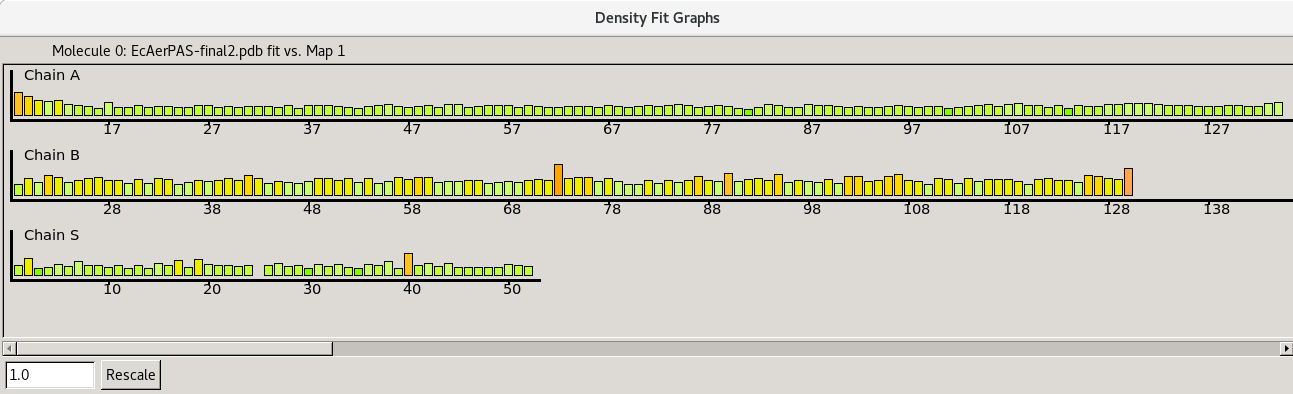
**

**Supplemental Figure 1.** Comparison of the two AerPAS-GVV molecules in the crystallographic asymmetric unit. (A) Chains A and B colored according to average residue B-factor. (B) MolProbity (1)-calculated correlation coefficients (CC) between Fc and 2mFo-DFc electron density maps for Chain A and Chain B, where Fc is the model-calculated structure factor, and Fo is the observed structure factor. <CC> for Chain A = 0.95; <CC> for Chain B = 0.87 (C) Coot calculated Density Fit Analysis (2) for Chain A, Chain B, and solvent molecules (Chain S).

**Supplementary Video 1.** 360^o^ rotation view of structural superpositions of PAS domains shown in Figure 6: *Escherichia coli* Aer-PAS-GVV PDBID: 8DIK (tan with cyan N-cap and orange C-terminal helix), *Methylococcus capsulatus* MmoS PAS domain PDBID: 3EWK (blue), *Azotobacter vinelandii* NifL PAS domain PDBID: 2GJ3 (purple), *Avena Sativa* Phototropin 1 LOV2 domain PDBID: 2V1A (green).

**References:**

1. Williams, C. J., Headd, J. J., Moriarty, N. W., Prisant, M. G., Videau, L. L., Deis, L. N., Verma, V., Keedy, D. A., Hintze, B. J., Chen, V. B., Jain, S., Lewis, S. M., Arendall, W. B., Snoeyink, J., Adams, P. D., Lovell, S. C., Richardson, J. S., and Richardson, D. C. (2018) MolProbity: More and better reference data for improved all-atom structure validation. *Protein Science* **27**, 293-315

2. Casanal, A., Lohkamp, B., and Emsley, P. (2020) Current developments in Coot for macromolecular model building of Electron Cryo-microscopy and Crystallographic Data. *Protein Science* **29**, 1069-1078
